# UAV rice panicle blast detection based on enhanced feature representation and optimized attention mechanism

**DOI:** 10.1186/s13007-025-01333-4

**Published:** 2025-02-04

**Authors:** Shaodan Lin, Deyao Huang, Libin Wu, Zuxin Cheng, Dapeng Ye, Haiyong Weng

**Affiliations:** 1 College of Mechanical and Electrical Engineering, Fujian Agriculture and Forestry University, Fuzhou, 350002 China; 2 School of Information and Intelligent Transportation, Fujian Chuanzheng Communications College, Fuzhou, 350007 China; 3 Fujian Key Laboratory of Agricultural Information Sensing Technology, Fuzhou, 350002 China; 4 College of Agriculture, Fujian Agriculture and Forestry University, Fuzhou, 350002 China

**Keywords:** Rice blast, Semantic segmentation, ConvNeXt, GAM, FocalTverskyLoss

## Abstract

**Background:**

Rice blast is one of the most destructive diseases in rice cultivation, significantly threatening global food security. Timely and precise detection of rice panicle blast is crucial for effective disease management and prevention of crop losses. This study introduces ConvGAM, a novel semantic segmentation model leveraging the ConvNeXt-Large backbone network and the Global Attention Mechanism (GAM). This design aims to enhance feature extraction and focus on critical image regions, addressing the challenges of detecting small and complex disease patterns in UAV-captured imagery. Furthermore, the model incorporates advanced loss functions to handle data imbalances effectively, supporting accurate classification across diverse disease severities.

**Results:**

The ConvGAM model, leveraging the ConvNeXt-Large backbone network and the Global Attention Mechanism (GAM), achieves outstanding performance in feature extraction, crucial for detecting small and complex disease patterns. Quantitative evaluation demonstrates that the model achieves an overall accuracy of 91.4%, a mean IoU of 79%, and an F1 score of 82% on the test set. The incorporation of Focal Tversky Loss further enhances the model's ability to handle imbalanced datasets, improving detection accuracy for rare and severe disease categories. Correlation coefficient analysis across disease severity levels indicates high consistency between predictions and ground truth, with values ranging from 0.962 to 0.993. These results confirm the model’s reliability and robustness, highlighting its effectiveness in rice panicle blast detection under challenging conditions.

**Conclusion:**

The ConvGAM model demonstrates strong qualitative advantages in detecting rice panicle blast disease. By integrating advanced feature extraction with the ConvNeXt-Large backbone and GAM, the model achieves precise detection and classification across varying disease severities. The use of Focal Tversky Loss ensures robustness against dataset imbalances, enabling accurate identification of rare disease categories. Despite these strengths, future efforts should focus on improving classification accuracy and adapting the model to diverse environmental conditions. Additionally, optimizing model parameters and exploring advanced data augmentation techniques could further enhance its detection capabilities and expand its applicability to broader agricultural scenarios.

## Introduction

Rice blast is one of the major diseases affecting rice, which can be classified into seedling blast, leaf blast, node blast, panicle blast, and neck blast. The occurrence and development of rice blast are closely related to meteorological conditions such as temperature, precipitation, and humidity, with humidity having the greatest impact [1]. If panicle blast occurs during the heading and flowering stages, it directly affects both yield and quality, and in severe cases, it can lead to total crop loss. The degree of damage varies depending on the variety, cultivation techniques, and climatic conditions. In epidemic years, yield reduction can generally range from 10 to 20%, and in severe cases, it can exceed 40 to 50% [2]. In recent years, significant progress has been made in monitoring and identifying rice blast in the field. However, these methods still have limitations that affect their application in early rice blast detection.

### Remote sensing and hyperspectral imaging methods

Aerial hyperspectral remote sensing images have been employed to evaluate infected areas of rice blast based on characteristic bands [3]. Similarly, field experiments have utilized hyperspectral imagers to obtain reflectance spectra of different rice leaf types (healthy, nitrogen-deficient, mildly infected, and severely infected) and analyze their spectral characteristics [4]. Various preprocessing methods combined with partial least squares discriminant analysis and principal component support vector machine methods have been used to construct rice leaf blast identification models, laying the foundation for large-scale remote sensing monitoring of rice blast [5]. However, these methods often face insufficient processing capabilities when dealing with large-scale hyperspectral data, which are complex and contain noise. This complexity requires sophisticated preprocessing to extract useful features, which can be computationally intensive and time-consuming [6].

### Machine learning and deep learning-based methods

Incorporating machine learning and deep learning into rice blast detection has brought some success. For example, deep convolutional neural networks like GoogLeNet have been used for rice panicle blast detection, where Inception modules capture multi-scale features of blast spots. These features are then combined through cascade fusion to improve detection accuracy. GoogLeNet further leverages its depth and width to learn high-dimensional features from hyperspectral images while employing a Softmax classifier for rice blast prediction modelling [7, 8]. Additionally, long short-term memory (LSTM) networks have been used to predict rice blast incidence based on historical disease and climatic data, with model performance evaluated by adjusting variables like temperature, relative humidity, and sunshine hours [9]. Deep learning models, such as GoogLeNet and LSTM, have demonstrated significant advantages in agricultural applications, including their ability to capture complex patterns and features in hyperspectral and UAV imagery. These models excel in identifying subtle disease characteristics and have contributed to advancements in precision agriculture. While challenges such as high computational complexity and substantial dataset requirements remain, this study addresses these issues by employing a lightweight and optimized ConvGAM model. By leveraging advanced multi-scale feature extraction and robust data augmentation techniques, the proposed approach reduces computational demands and enhances performance even with limited datasets. This ensures scalability and applicability in real-time, large-scale agricultural scenarios. Additionally, recent studies have demonstrated the effectiveness of deep learning in agricultural disease detection. Such as a lightweight attention-based network that achieved state-of-the-art performance in detecting crop diseases with reduced computational costs, highlighting the transformative potential of deep learning in real-time agricultural applications [10].

### Field telemetry and UAV-based detection

Field telemetry technology has been applied to provide early warnings for rice blast occurrence, offering valuable guidance in reducing yield loss and fungicide use [11]. Meanwhile, UAV-based hyperspectral platforms have been used to obtain canopy data for rice neck blast at various disease levels. Spectral data with different treatments were used as inputs into random forest models for disease grading and monitoring. The associations of these spectral features were explained in combination with rice physiology, supporting quantitative remote sensing monitoring and early warning grading of neck blast [12–14]. Additionally, multispectral data from infected fields were collected using UAVs equipped with six-channel multispectral cameras, where variance analysis was employed to select 19 characteristic spectra for rice blast classification [15]. Although UAV-based methods advance quantitative remote sensing monitoring, they face sensitivity to environmental conditions, where factors like fluctuating light intensity and atmospheric conditions can significantly affect image quality and detection accuracy. Additionally, they often involve high computational costs due to complex data processing and analysis, limiting their real-time application.

### Handling data imbalance

Several studies have focused on the challenge of data imbalance in rice blast detection. For instance, a model for leaf blast detection was developed using leaf spectral ratios and support vector machines (SVMs) to classify rice blast at the leaf scale [16]. Similarly, regional-scale rice panicle blast classification models using stepwise linear discriminant analysis, SVMs, and neural networks were compared, revealing that even with accurate identification, imbalanced data could lead to biased results [17]. In another study, naturally infected rice leaves were collected to establish a multi-classification SVM model for early rice blast detection, highlighting the difficulty in accurately identifying less common disease stages due to the limited sample numbers [18].The data imbalance problem makes it challenging for models to generalize well across different disease stages, thereby reducing overall detection accuracy, especially for early-stage disease identification.

### Integrated models and weather feature-based predictions

More recent methods have incorporated deep learning for identifying various rice diseases, including rice blast, false smut, neck blast, sheath blight, bacterial leaf streak, and brown spot [20]. Integrated models played a significant role in enhancing the prediction capabilities, particularly when combined with meteorological data. A system was developed to rank 15 weather features using ensemble methods, identifying average visibility, rainfall, sunshine hours, maximum wind speed, and rainy days as the most effective predictors of rice blast [19, 21]. Another method, RiceNet, was introduced for identifying rice panicle blast and rice blast, utilizing YoloX to detect rice disease spots and a twin network to classify the disease severity into four categories [22]. While these methods have improved prediction accuracy, they still require highly specialized datasets and face challenges related to data imbalance when predicting disease severity levels, limiting their applicability across different regions and growth stages.

In this study, we propose a novel rice blast detection method that aims to address the limitations of the above-mentioned approaches. Unlike traditional hyperspectral methods, which struggle with high-dimensionality and noise, our approach leverages the ConvNeXt-Large backbone to extract fine-grained, multi-scale features, enabling precise detection of subtle disease patterns under challenging UAV imaging conditions. To address data imbalance, we incorporate advanced data augmentation to diversify training samples and employ the Focal Tversky Loss function to improve the detection of rare and severe disease categories. These innovations ensure robust feature extraction and reliable generalization across varying disease severities and environments.

Its main contributions are as follows:

1. Enhancing Feature Extraction: ConvNeXt [23] effectively extracts detailed features from the images, which are crucial for identifying small and complex disease patterns.
2. Improving Attention Mechanism: GAM [24] enhances the model's focus on significant regions, improving the accuracy of disease detection.
3. Handling Imbalanced Data: Focal Tversky Loss [25], focusing on difficult-to-classify samples, ensures that the model performs well even with imbalanced datasets.

## Material and method

### Experimental site

**Fig. 1:**
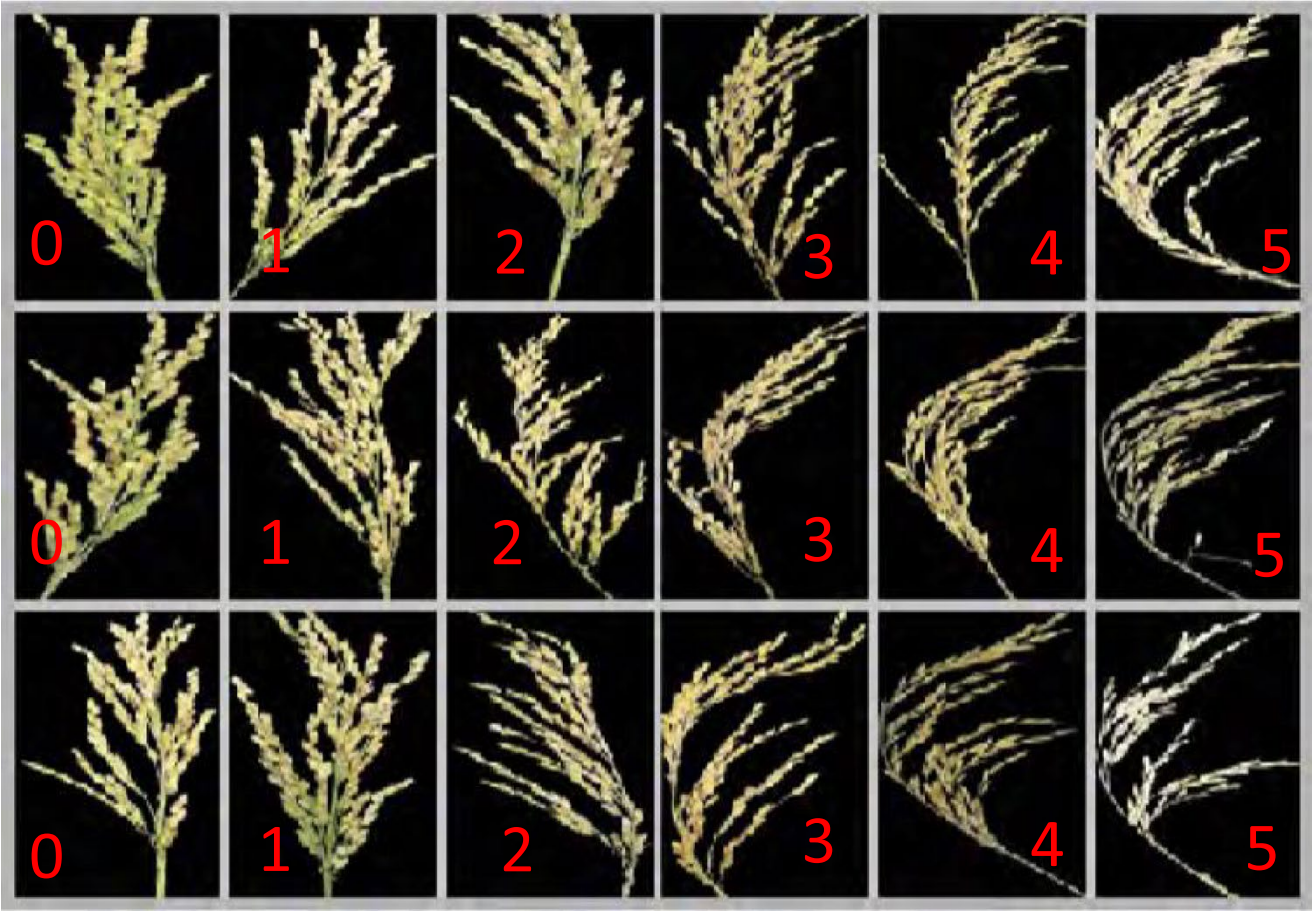
Diseased rice panicles. Each sub-image is labeled with a number indicating the disease severity level: “0” for healthy, “1” for mild infection, “2” for moderate infection with noticeable shrinkage and bending, “3” for more severe shrinkage, “4” for significant grain loss and bending, and “5” for extreme cases where panicles are almost entirely shriveled

The experimental site is located in the rice breeding demonstration base in Chaidi Town, Shanghang County, Longyan City, China, with the longitude of 116.575°E and the latitude of 25.02°N. The dibble sowing method is used for sowing, with each variety planted in an area of 0.02 square meters, and over 1000 rice varieties with different resistance to rice blast are planted. Around each plot, an inducible variety “Minghui 86” is planted as a guard row. The seedlings are grown under moist conditions with a water depth of approximately 10–15 cm to ensure adequate water supply during the seedling stage. However, during the data collection period at the ripening stage, the water depth is reduced to a shallow level. The fertilization amount follows the local field standards. N, P, and K fertilizers are applied in the form of Ca(H_2_PO_4_)_2_∙H_2_O and KCl, with application rates of 162.6 kg/ha, 90.6 kg/ha, and 225.0 kg/ha, respectively [26]. To obtain different degrees of disease severity for model training, the model is trained on three Tesla V100 GPUs. The batch size is set to 6 to obtain the pre-training weight. The initial learning rate is set to 1 × 10^–3^ and is decayed to1 × 10^–5^ over 3000 steps. The natural field induction process includes: (1) selecting rice varieties with different levels of resistance to rice blast, (2) ensuring the environmental conditions (humidity and temperature) at the experimental site are conducive to the growth and spread of rice blast, and (3) monitoring the rice plants during specific growth stages to observe the onset and development of rice blast symptoms. By following this method, diseased rice panicles were successfully obtained (as shown in Fig. 1).

### UAV image collection

**Fig. 2:**
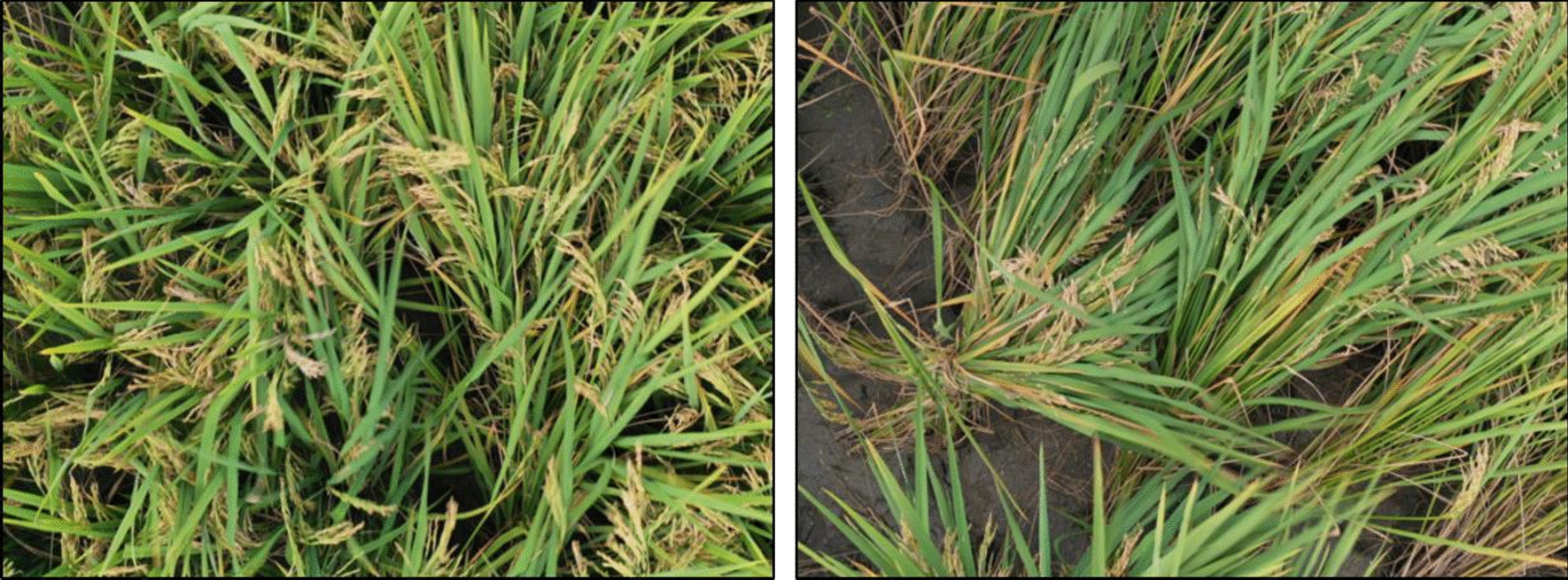
UAV images of diseased rice panicles. High-resolution RGB images of diseased rice panicles were collected using a UAV during optimal weather conditions, ensuring comprehensive coverage and image quality for accurate analysis and model training

A commercial UAV (DJI Mavic 2 Pro, manufactured by Shenzhen DJI Sciences and Technologies Ltd.) was used to collect high-resolution RGB images (5472 × 3648 pixels) during the ripening stage in October 2023, from 14:00 to 16:00 local time. The weather was clear, with a temperature of 30 °C, the humidity of 72%, and no wind, which is the optimal condition for image acquisition. The UAV was flown at an altitude of 5 m, with a camera exposure time of 0.2 ms, resulting in a ground resolution of 1 cm/pixel. To ensure comprehensive coverage and high-quality data [27], the flight path was meticulously planned with 60% forward overlap and 75% side overlap [28]. This setup minimized blind spots and ensured sufficient image overlap for accurate stitching and analysis. In total, 1175 high-resolution UAV images of diseased rice panicles (as shown in Fig. 2) were captured, providing a robust dataset for subsequent analysis and model training. The dataset is categorized into six severity levels (0–5) based on the progression of rice blast. The distribution is notably imbalanced, with the majority of samples classified into lower severity levels: 35% as Level 0 (Healthy), 25% as Level 1 (Very Mild), and 15% as Level 2 (Mild). Higher severity levels are underrepresented, with 12% at Level 3 (Moderate), 8% at Level 4 (Severe), and only 5% at Level 5 (Extreme). We divide the dataset into a 8:1:1 ratio, with the training set consisting of 939 images, the validation set of 118 images, and the test set of 118 images.

### Model evaluation

**Fig. 3:**
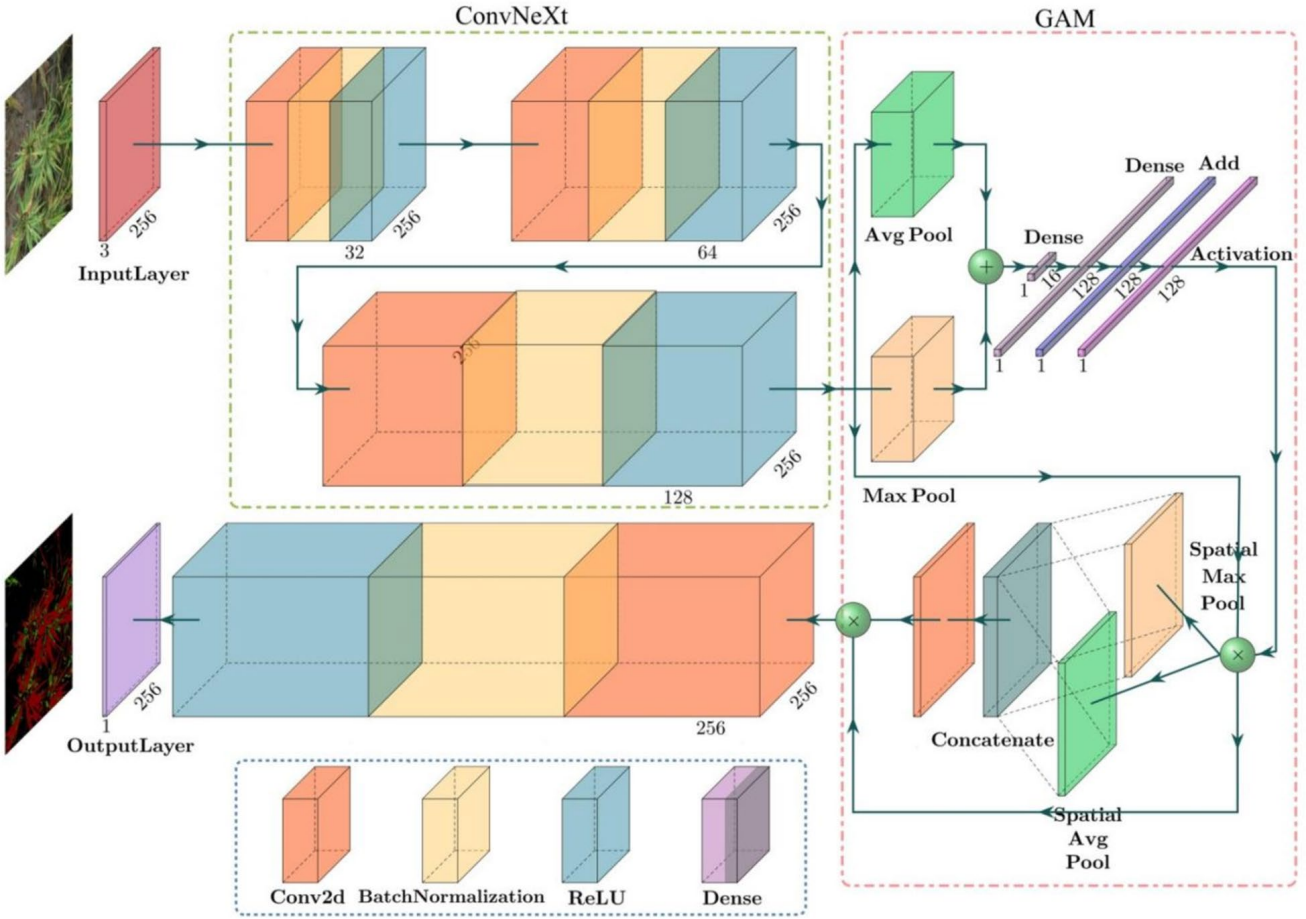
ConvGAM model framework. The model is mainly composed of two blocks. ConvNeXt extracts detailed multi-scale features from the input images, enhancing the model’s ability to identify complex disease patterns, while the Global Attention Mechanism (GAM) dynamically concentrates on significant regions in the images, improving detection accuracy by highlighting areas most affected by the disease

This study focuses on detecting rice panicle blast disease. For the rice planted in the experimental base, a large number of raw RGB images of diseased rice panicles are collected by UAVs flying at heights of 5 m. By analyzing these images and detecting the diseased areas of the panicles, we aim to assess the growth status and yield of the rice. To achieve accurate panicle blast detection, instance segmentation must be performed first. We employ an enhanced multi-scale attention mechanism (ConvGAM) semantic segmentation model (as shown in Fig. 3), which uses the raw UAV-collected images as input. The model mainly consists of four parts: feature extraction, feature enhancement, classification and segmentation, and loss function optimization. The segmentation task is divided into two stages:

1. Feature extraction and enhancement

This stage uses a deep convolutional neural network (such as ConvNeXt [23]) to perform multi-level, multi-scale feature extraction on the input images. The features are enhanced through a Global Attention Mechanism (GAM) to improve the robustness and discriminative power of the feature representations.

(2) Classification and segmentation

In this stage, the high-level features obtained from the feature extraction process are used for pixel-level classification, resulting in the final segmentation output.

The Fig. 3 illustrates the structure of the ConvGAM (ConvNeXt Network and Global Attention Mechanism) model framework. The entire model framework is divided into several parts. First, the image enters the model through the input layer, with an input image size of (1024 × 1024 × 3). The UAV captures high-resolution images, often with dimensions like 5472 × 3648 pixels, which are resized or downsampled to (1024 × 1024 × 3) to match the input size required by the model. This resizing is a standard pre-processing step performed using image processing libraries such as OpenCV or PIL, ensuring uniform dimensions for model input. The “3” in (1024 × 1024 × 3) refers to the RGB color channels, which remain unchanged during resizing. This process is essential for deep learning models like ConvNeXt, which require fixed input sizes to maintain consistency in the network's operations.

Next, the input image passes through multiple convolution layers (Conv2d), each followed by Batch Normalization, ReLU activation, and Dropout layers. The diagram shows three convolution layers, with the feature map depth gradually increasing to 32, 64, and 128, respectively. The feature maps then undergo both spatial pooling and global pooling to extract features. Spatial pooling includes Max Pooling and Average Pooling, while global pooling processes the entire feature map. Subsequently, the feature maps are concatenated after spatial pooling, combining both local and global features. The Global Attention Mechanism (GAM) is used to further enhance the feature representations.GAM uses an attention mechanism to allocate different weights to various parts of the feature map, giving higher weights to regions important for detecting diseases, like infected rice panicles, while assigning lower weights to less relevant regions. This process amplifies the key features in the important areas, making the model more sensitive to the visual characteristics of diseased regions and less influenced by irrelevant background information. GAM operates globally across the entire image, ensuring that significant features throughout the image are emphasized, helping the model capture both large-scale patterns and small details related to disease. In summary, GAM enhances the model’s ability to focus on critical parts of the image, improving its capacity to differentiate diseased areas from healthy ones by highlighting the most relevant features.

The enhanced feature maps are input into dense layers and activated by an activation function, and then the classification results is generated. The final output feature map size is (224 × 224 × 3), the same as the input image size, which is used to generate the segmentation results. The output layer produces the final segmented image, marking the diseased areas of the rice panicles. The lower part of the diagram shows the specific structure of each component, including the combination of convolution layers, batch normalization, ReLU activation, and dropout layers. By integrating convolutional neural networks and the global attention mechanism, the entire model effectively improves the detection and segmentation accuracy of rice panicle blast.

### ConvGAM

#### BackBone

**Fig. 4:**
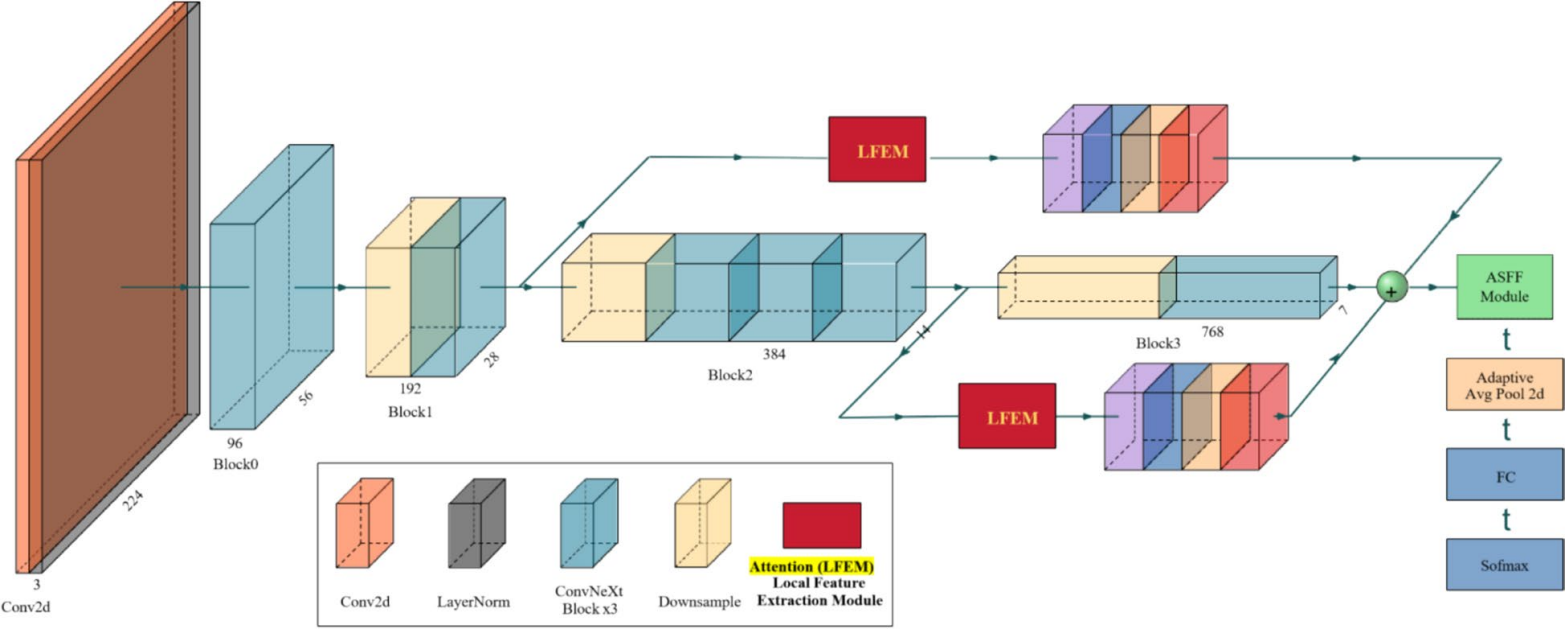
ConvNeXt network. The highlighted ‘Attention (LFEM)’ block dynamically adjusts feature map weights through channel and spatial dimensions, focusing on key areas using GELU activation

For the detection of panicle blast, we require a backbone network that can efficiently and accurately extract image features to precisely identify and segment diseased spots in rice panicles. ConvNeXt (as shown in Fig. 4) has significantly improved the feature extraction capability and accuracy while maintaining low computational complexity and parameter size through a series of innovative designs,including Modernized Convolutional Layer Designs, Depthwise Convolutions, and Transformer-Like Layer Scaling, etc.Therefore, ConvNeXt is an ideal choice for our panicle blast detection. ConvNeXt is an optimized convolutional neural network architecture that progressively extracts multi-scale features of the input image through layer-by-layer convolution, layer normalization, and non-linear activation functions. Specifically, the initial layers of the network include Conv2d and LayerNorm layers, which are responsible for preliminary convolution operations and normalization processing, respectively. Subsequently, a series of convolutional layers gradually reduce the size of the feature maps but increase the number of channels to extract deeper features [23].

ConvNeXt adopts depth-wise convolution, significantly reducing computational load and parameter size while maintaining efficient feature extraction capability [23]. This allows it to better capture detailed features in rice panicle images. By introducing an attention mechanism that adjusts the weights of the feature maps dynamically through both channel and spatial dimensions, the model can adjust the convolution kernel size and use a more suitable activation function (GELU) to better focus on the key areas affected by panicle blast. ConvNeXt enhances feature representation capabilities while still maintaining high accuracy [23], making it particularly effective for panicle blast detection. There are four versions of the ConvNeXt network, as shown in Table 1, with the specific version used in this study being ‘ConvNeXt-Large’.

**Table 1:** Four versions of the ConvNeXt network

ConvNeXt network	Channels	Blocks
ConvNeXt-Tiny	(96, 192, 384, 768)	(3, 3, 9, 3)
ConvNeXt-Small	(96, 192, 384, 768)	(3, 3, 27, 3)
ConvNeXt-Base	(128, 256, 512, 1024)	(3, 3, 27, 3)
ConvNeXt-Large	(192, 384, 768, 1536)	(3, 3, 27, 3)

The convolution block structure of ConvNeXt primarily consists of depth-wise convolution and point-wise convolution. Its core mathematical expressions are as follows:

1 $${Y}_{i}={\sum }_{j=1}^{k\times k}{X}_{i+j-1}\cdot {W}_{j}+b$$

where *Y*_*i*_ is the value at the *i* position of the output feature map, *X*_*i*+*j−*1_ is the value at the *i* + *j−1* position of the input feature map, *W*_*j*_ is the j weight of the convolution kernel, and *b* is the bias value. After each convolutional layer, Layer Normalization (LayerNorm) and the GELU activation function are applied:

2 $${Y}_{norm}=\frac{{Y}_{i}-\mu }{\sqrt{{\sigma }^{2}+\epsilon }}$$

3 $${Y}_{GELU}={Y}_{norm}\cdot \Phi ({Y}_{norm})$$

where *μ* and *σ* are the mean and standard deviation of the feature map, respectively. *ϵ* is a small number to prevent division by zero, and *Φ* is the cumulative distribution function of the standard Gaussian distribution, and *Y*_*norm*_ is the normalized output of the feature map, *Y*_*GELU*_ is the output processed using the Gaussian Error Linear Unit (GELU) activation function.

Through the above convolution operations, ConvNeXt-Large can efficiently extract and process image features, providing high-quality feature maps for subsequent attention mechanisms and segmentation tasks. We chose ConvNeXt-Large as the backbone network because it can achieve high-precision feature extraction and segmentation for panicle blast detection, ensuring that the diseased areas are accurately identified. ConvNeXt-Large has a high number of channels and blocks, allowing it to capture more detailed features and improve the detection accuracy. Additionally, ConvNeXt-Large can extract rich multi-scale features, which is important for handling variations in shooting angles and panicle heights. It also improves the robustness of the model in practical applications.

### Global attention mechanism

**Fig. 5:**
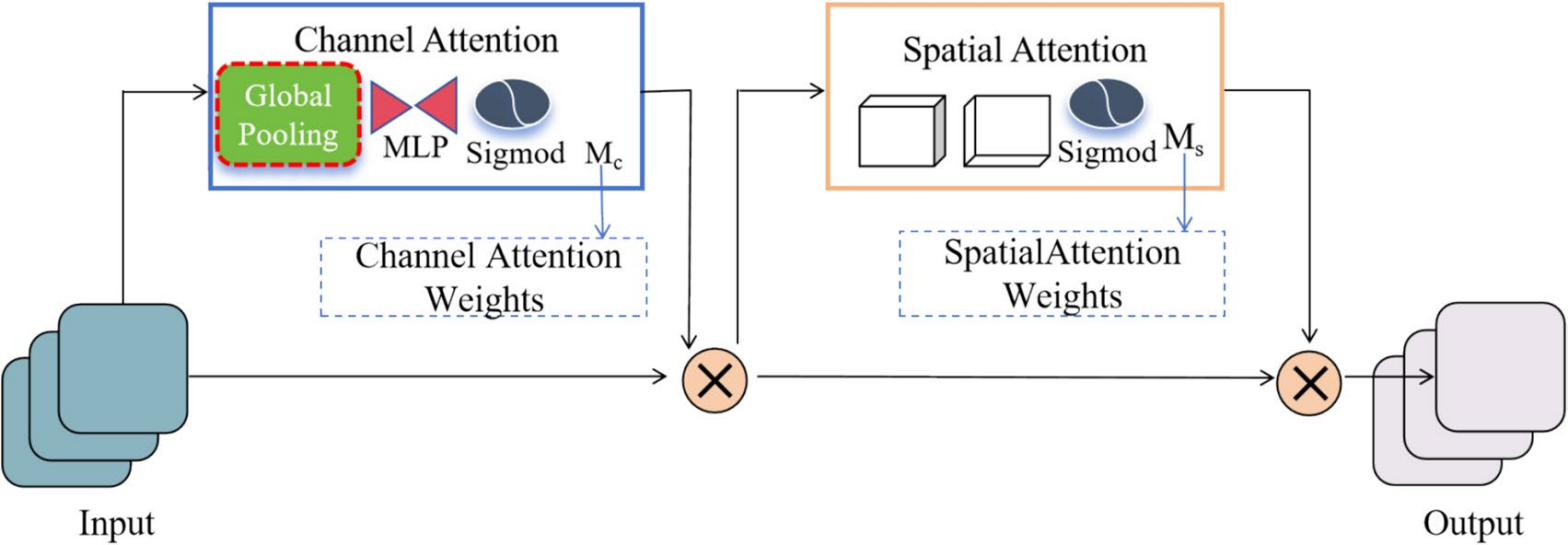
The structure of GAM. The figure illustrates the structure of the GAM, where the channel attention module uses global pooling and a two-layer MLP to amplify channel-spatial dependencies, while the spatial attention module integrates spatial information through convolutional layers, with Sigmoid processing applied to both modules to generate attention weights

To further enhance feature representation capability, we introduced GAM (Global Attention Mechanism). GAM dynamically adjusts the weights of the feature maps through channel and spatial dimensions, allowing the model to better focus on key areas affected by panicle blast. The GAM attention mechanism is designed to improve the performance of deep neural networks, which is consists of two main modules: the channel attention module and the spatial attention module. The structure of GAM is shown in Fig. 5.

In GAM, the channel attention module uses a three-dimensional arrangement to retain information and amplifies cross-dimensional channel-spatial dependencies through a two-layer MLP (Multilayer Perceptron). During this process, the feature map is first transformed in dimension, then input into MLP, and finally transformed back to its original dimensions. Sigmoid processing is performed to output the channel attention weights. The spatial attention module focuses on spatial information by using two convolutional layers to integrate spatial information. It obtains the reduction ratio from the channel attention module, uses convolution to reduce or increase the number of channels, and finally output the spatial attention weights through Sigmoid processing. The specific working steps are as follows:

1. Global Pooling: Global pooling is performed on the input feature map to obtain the global feature vector for the channel dimension.
  4 $$z=\frac{1}{H\times W}\sum_{i=1}^{H}\sum_{j=1}^{W}X(i,j)$$

where *X* represents the input feature map, and *z* is the vector obtained after global pooling, and (*i,j)* is indices for the height and width, respectively, and *X(i,j)* is the feature value at spatial position (*i,j*) for each channel, and $\frac{1}{H\times W}$ is the normalization factor, representing the total number of spatial positions over which the sum is taken.

(2) MLP Processing: Process the vector through a two-layer MLP (Multilayer Perceptron) to amplify the dependencies between channels.where *W*_*1*_ and *W*_*2*_ are the weight matrices of the MLP, and z is the input vector, and *σ* is the Sigmoid function, and $\delta$ represents the ReLU (Rectified Linear Unit) activation function,and z^’^ is the output vector after processing through the MLP layers.
  5 $${z}^{\prime}=\sigma ({W}_{2}\cdot \delta ({W}_{1}\cdot z))$$

(3) Reshape and Weight Application: Reshape the processed feature vector back to its original dimensions and multiply it with the input feature map element-by-element to obtain the channel-enhanced feature map.where *X* represents the input feature map, and z^’^ is the channel attention vector obtained after MLP processing, and ⊙ denotes element-wise multiplication, and *X*^*’*^ is the output feature map with enhanced channels.
  6 $$X^{\prime} = X \odot z^{\prime} )$$

The spatial attention module is used to enhance the information of the feature map in the spatial dimension. The specific steps are as follows:

1. Convolution Processing: Process the channel-enhanced feature map through two convolutional layers to integrate spatial information.
  7 $$X_{s} = \sigma (W_{s} *X^{\prime})$$

where *W*_*s*_ represents the convolution kernel, and * denotes the convolution operation, and *σ* is the Sigmoid function, and *X*^*’*^ represents the input feature map, and *X*_*s*_.

(2) Weight Application: Multiply the spatial attention weights with the channel-enhanced feature map element-by-element to obtain the final enhanced feature map.where *X*^*’*^ is the channel-enhanced feature map obtained from the previous step, and *X*_*s*_ represents the spatial attention weights, and *X*^*’’*^ is the final enhanced feature map after applying spatial attention, and ⊙ denotes element-wise multiplication.
  8 $${X}^{{\prime}{\prime}}={X}^{\prime}\odot {X}_{s}$$

The advantage of GAM lies in its fine-grained feature extraction capability. As the diseased areas of panicle blast are often small and complexly distributed, GAM can effectively capture these fine-grained features through its channel and spatial attention modules, thereby improving detection accuracy. The design of GAM enhances the flow of information by allowing better movement of channel and spatial information within the feature map, enabling more accurate identification and separation of diseased areas from the background. GAM can be applied to complex agricultural scenarios, typically characterized by intricate background information. GAM can suppress background noise and highlight target areas, thus improving the detection performance in the challenging environments. By combining ConvNeXt-Large with GAM, we can leverage the strengths of both to enhance the performance of panicle blast detection. ConvNeXt-Large, as the backbone network, is responsible for extracting fundamental features from the input images, while the GAM module enhances these features in both channel and spatial dimensions, further improving the feature representation capabilities.

### Focal Tversky Loss

Focal Tversky Loss is an extension of traditional Dice Loss and Tversky Loss [25], which is designed to address class imbalance issues and enhance the model's focus on hard-to-classify samples. Tversky Loss is an extension of Dice Loss that allows more flexible control over the weights of false positives (FP) and false negatives (FN). The formula is as follows:

9 $$Tversky \,Index=\frac{TP}{TP+\alpha \cdot FN+\beta \cdot FP}$$

Among them, TP is True Positive, the number of correctly predicted positive samples. *FN* is False Negative, the number of actual positive samples incorrectly predicted as negative. *FP* is False Positive, the number of actual negative samples incorrectly predicted as positive. *α* and* β* are weight parameters that control the weights of *FN* and *FP*, respectively.

There may be class imbalance in panicle blast detection. By adjusting the *α* and* β* parameters, Focal Tversky Loss can better handle the class imbalance, so that the model can perform well on minority classes. By introducing the* γ* parameter, Focal Tversky Loss can make the model focus more on hard-to-classify samples, enhancing the model's robustness and accuracy. When calculating loss, Focal Tversky Loss can balance the impact of false positives and false negatives, allowing the model to perform better in fine segmentation tasks by reducing the false and missed detections. Focal Tversky Loss further incorporates the idea of Focal Loss to enhance the model's focus on hard-to-classify samples. Assuming that the input consists of the predicted mask *P* and the ground truth mask *G*, both with shape *(N,H,W)*, where N is the number of samples, and *H* and *W* are the height and width of the images, respectively, the calculation formulas for Tversky Index and Focal Tversky Loss are as follows:

10 $$Tversky \,Index=\frac{\sum P\cap G}{\sum P\cap G+\alpha \cdot \sum P\backslash \text{G}+\beta \cdot G\backslash P}$$

where *P* represents the predicted mask value, *G* represents the ground truth mask value,* α* is a parameter that controls the weight of false positives, *β* is a parameter that controls the weight of false negatives, *P ∩ G* represents the intersection of the predicted and ground truth masks (True Positives),* P∖G* represents the false positives, and *G∖P* represents the false negatives.

11 $$Focal \,Tversky \,Loss={(1-Tversky\, Index)}^{\gamma }$$

where *γ* is a parameter that adjusts the focus on hard-to-classify samples. These formulas allow Focal Tversky Loss to address class imbalance issues effectively. By adjusting the parameters *α, β,* and *γ,* the model performs well on minority classes and enhances the focus on difficult-to-classify samples. This improves the model's robustness and accuracy, particularly in fine segmentation tasks by balancing the impact of false positives and false negatives, thereby reducing both false and missed detections.

### Evaluation metrics

In order to assess the model’s predictive capabilities, the following evaluation metrics are taken into account [29, 30]:

12 $$\begin{array}{c}Accuracy= \frac{TP}{TP+TN+FP+FN}\end{array}$$

13 $$\begin{array}{c}Precision= \frac{TP}{TP+FP}\end{array}$$

14 $$\begin{array}{c}Recall= \frac{TP}{TP+FN}\end{array}$$

15 $$\begin{array}{c}F1 score= \frac{2\times Precision\times Recall}{Precision+Recall}\end{array}$$

16 $$\begin{array}{c}Average \,accuracy= \frac{1}{N}\sum_{i=1}^{N}\frac{{TP}_{i}}{{TP}_{i}+{TN}_{i}+{FP}_{i}+{FN}_{i}}\end{array}$$

Among them, *TP* is true positives, *TN* is true negatives, *FP* is false positives and *FN* is false negatives. As shown in formula (12–15), accuracy is a metric that measures the proportion of correct predictions out of all the predictions made. Precision is calculated as the proportion of correct positive predictions out of all the predicted positives. Recall means the ratio of correct positive predictions out of all the actual positive instances. And F1 score, as an additional measure of the accuracy for a classification problem, is a metric that provides a balance measure of precision and recall. It can be written as the harmonic mean of these two metrics. Average accuracy is defined as the mean of the accuracies across all classes, which is calculated as follows: *N* represents the total number of classes, and *TP*_*i*_*, TN*_*i*_*, FP*_*i*_*, FN*_*i*_ are the true positives, true negatives, false positives, and false negatives for the *i*-th class, respectively.

### Calculation method for diseased area

In this study, the diseased area is calculated based on the semantic segmentation results. The HSV color range is defined to identify different diseased color regions. The classification of disease levels for panicle blast is based on the International Rice Research Institute standards [31], as shown in Table 2. The grading of panicle blast is divided into levels 0–5, with level 0 representing no disease, and levels 1–5 representing various degrees of infection. The grading criteria for diseased levels are shown in Table 2. The specific HSV color ranges can be adjusted according to the actual colors in the images.

**Table 2:** Panicle blast levels

Level	HSV thresholds	Rule
0	H: 0–10, S: 70–100, V: 50–100	No disease
1	H: 50–70, S: 30–60, V: 40–90	< 5% (Infection in a few branches)
2	H: 60–80, S: 20–50, V: 50–90	5.1–20% (Infection in about half of the branches)
3	H: 80–100, S: 40–70, V: 60–100	20.1–50% (Infection in the panicle neck or main axis, some empty grains)
4	H: 100–120, S: 50–80, V: 70–100	50.1–70% (Infection in the panicle neck, most grains are empty)
5	H: 30–50, S: 80–100, V: 50–90	70.1–100% (Infection in the panicle neck, resulting in white panicles)

The area calculation is based on the number of pixels within each color range. Assuming the image resolution is width × height, the area of the color region can be expressed as:

17 $${Area}_{color\_id}={\sum }_{x=1}^{width}{\sum }_{y=1}^{height}f\left\{mask\left(x,y\right)=color\_id\right\}$$

where *f{⋅}* is an indicator function that returns 1 if the condition is true, otherwise 0,and color_id is the identifier for the specific color range used to classify the disease level, *Area*_*color_id*_ represents the area of the region with a specific color identified bycolor_id, and width and height are the dimensions of the image, representing the number of pixels along the width and height of the image,and mask(x, y) represents the color value of the pixel at position (x, y) in the image.

The calculated area is in pixels. In practical applications, pixels should be converted to conventional area units (e.g., square meters). Thus, it is necessary to know the image resolution and the physical size covered by the image. The specific steps are as follows:

1. Determine the image resolution: This is usually determined by the camera parameters, typically in units like pixels/cm or pixels/m. If the image is captured by a drone, the physical area covered by the image can be calculated based on the drone's altitude and the camera's field of view.
2. Calculate the actual area represented by each pixel: Assuming that the drone's flight height during image capture is *H* meters, the camera's field of view (horizontal angle) is *θ* degrees, and the image width is W pixels. Based on geometric relationships, the actual width of the image *W*_*real*_ can be calculated as follows:
  18 $${W}_{\text{real}}=2\times H\times \text{tan}\left(\frac{\theta }{2}\right)$$

The area of a single pixel *A*_*pixel*_ is

19 $${A}_{pixel}=\frac{{W}_{real}}{W}\times \frac{{H}_{real}}{H}$$

*H*_*real*_ is the actual height of the image. The total area in square meters is obtained by multiplying the number of pixels in the diseased area by *A*_*pixel*_ [32]_*.*_

The method of calculating the diseased area based on color segmentation plays an important role in the detection of panicle blast disease. By accurately identifying and calculating the area of diseased spots, it provides scientific data support for crop disease monitoring and management. This calculation method is used to derive the ground truth data (validation data) for the models, ensuring accurate model evaluation and training. The following is the pseudocode for calculating the area of the diseased region.

**Algorithm 1:**
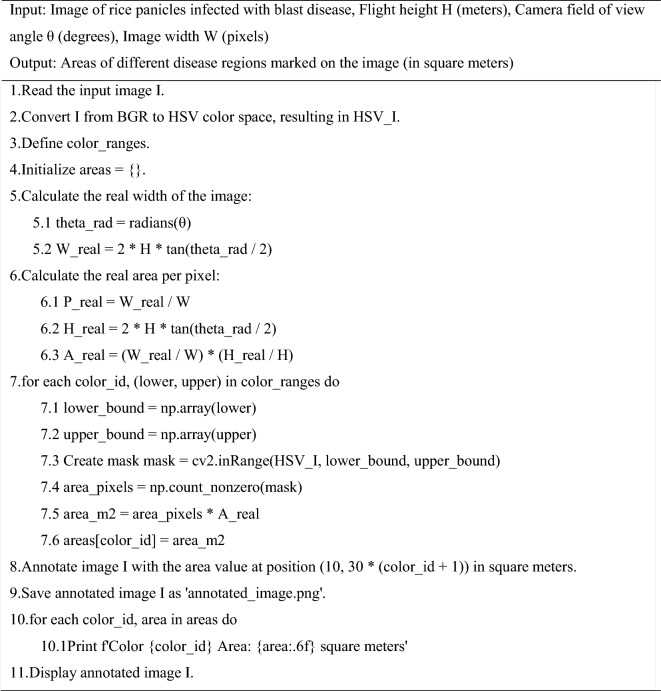
Computation of blast disease area in rice panicles

This pseudo-code describes the process of calculating the area of different disease regions in an image of rice panicles infected with blast disease. It involves image preprocessing, color ranges definition, calculation of each color range, results saving and displaying, and annotation of the image with the calculated areas.

## Result

In this study, we propose an enhanced feature extraction global attention mechanism semantic segmentation model—ConvGAM, which is used for detecting rice panicle blast disease. We conduct experimental analyses to evaluate the impact of various attention mechanisms on the model's efficacy.

### Training gradient analysis

**Fig. 6:**
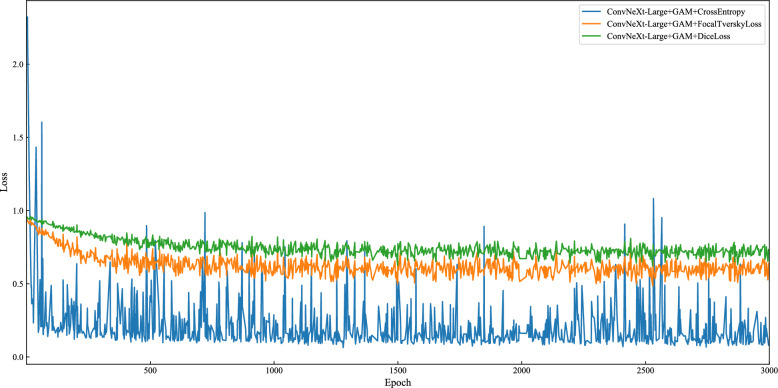
ConvGAM Training Gradient. The figure shows that Focal Tversky Loss achieves a lower and more stable loss compared to Dice Loss and Cross-Entropy, indicating better overall performance and faster learning during the training process, with Cross-Entropy exhibiting significant fluctuations in the later stages

ConvGAM experiments with three loss functions—Focal Tversky Loss, Dice Loss, and Cross-Entropy—to evaluate their impact on model performance (as shown in Fig. 6). These loss functions were chosen based on their potential to handle imbalanced datasets and to improve segmentation accuracy, particularly in semantic segmentation tasks. The experimental results from our dataset (as illustrated in Fig. 6) show that Focal Tversky Loss provides more stable gradient behavior during training, leading to smoother convergence. However, it is important to note that these results are based on a single dataset, making them preliminary. To confirm the broader applicability of these findings, further validation across additional datasets is necessary. Previous research, particularly in the field of medical image segmentation on imbalanced datasets, supports the notion that Focal Tversky Loss reduces gradient fluctuations, which contributes to more stable convergence. This correlation between gradient stability and effective convergence suggests that Focal Tversky Loss could offer similar benefits in other contexts, though further testing is required to verify this [33].

From the robustness perspective, due to the smoother gradient changes, the model trained with Focal Tversky Loss is more robust to noise and outlier data points during training. This means the model can better handle the complex and imbalanced data in reality, particularly when dealing with imbalanced classes or small sample data. Although Cross-Entropy can quickly reduce loss in the initial stages, its gradient fluctuates significantly in the later stages, showing its sensitivity to noise and outlier data, which may affect the final stability of the model. Focal Tversky Loss, by introducing the gamma parameter, can pay more attention to hard-to-classify samples. The gamma parameter in Focal Tversky Loss adjusts the model’s focus during training by assigning greater weight to hard-to-classify samples. This means that during the learning process, samples that the model struggles with (those that are harder to correctly predict) are given more importance in the loss function. By reducing the impact of easily classifiable examples and enhancing the influence of difficult samples, gamma helps the model refine its ability to distinguish challenging cases, leading to more robust training and better performance, particularly on minority classes or ambiguous regions in the image. Dice Loss and Cross-Entropy lack this specific focus on hard-to-classify samples, and therefore may not perform as well as Focal Tversky Loss when dealing with these samples.

From Fig. 6, it can be observed that Focal Tversky Loss is able to achieve a lower loss value in a shorter amount of time and maintain a low loss during the stable phase. This indicates that the model not only learns quickly in the initial stages but also maintains excellent performance in the later stages. Although Dice Loss can reach a relatively stable state during training, its loss value is high, indicating that its overall performance may not be as good as Focal Tversky Loss. Cross-Entropy quickly reduces the loss in the initial stages but exhibits greater fluctuations in the later stages, resulting in poorer stability.

Overall, Focal Tversky Loss provides smoother gradient changes during training, enhancing the model's stability and robustness. This allows the model to more effectively handle imbalanced data and hard-to-classify samples. Compared to Dice Loss and Cross-Entropy, this model has better overall performance. Although Dice Loss can maintain a certain level of stability, its higher loss value affects the final outcome. Although Cross-Entropy can converge quickly, it shows significant gradient fluctuations in later stages, and it is more sensitive to noise and outlier data, which impacts the model's stability.

### Optimal model combination

**Fig. 7:**
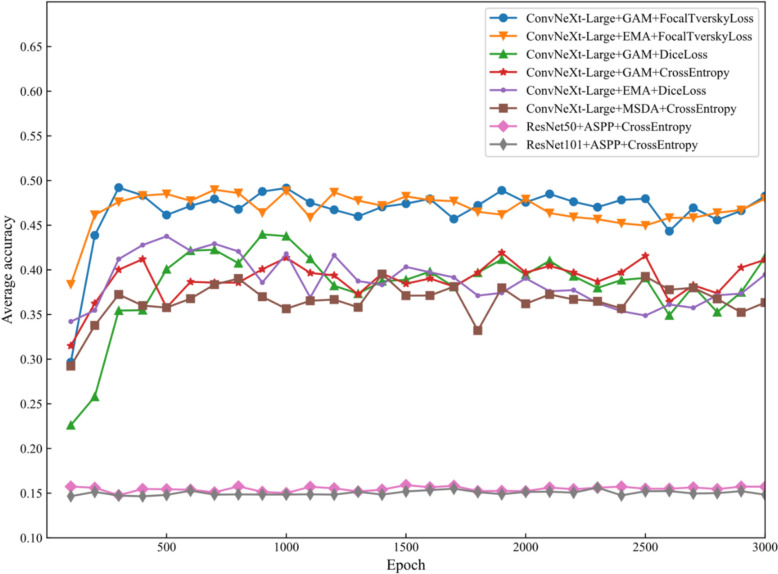
Comparison of average accuracy of models. The figure shows that the best-performing combinations, ConvNeXt-Large with GAM and FocalTverskyLoss, as well as ConvNeXt-Large with EMA and FocalTverskyLoss, achieve the highest and most stable accuracy, while ResNet architectures with CrossEntropy perform the worst, indicating their unsuitability for this task

To evaluate the average accuracy performance of different model combinations in the task of detecting panicle blast disease, we select various combinations of models and loss functions. The aim is to compare these combinations in handling imbalanced data and complex tasks. These combinations include different network architectures (ConvNeXt-Large and ResNet), different attention mechanisms (GAM, EMA, MSDA), and different loss functions (Focal Tversky Loss, Dice Loss, Cross-Entropy). Through comparative analysis, we aim to identify the optimal model combination. Figure 7 shows the average accuracy over epochs for various model and loss function combinations used in the task of detecting panicle blast disease. The x-axis represents the number of training epochs, while the y-axis represents the average accuracy. Each curve represents a different combination of network architecture, attention mechanism, and loss function.

The best-performing combinations are ConvNeXt-Large + GAM + FocalTverskyLoss and ConvNeXt-Large + EMA + FocalTverskyLoss, which exhibit the highest and most stable accuracy. Combinations of ConvNeXt-Large with DiceLoss and CrossEntropy show moderate performance but greater fluctuations. The poorest ones are the ResNet architectures with CrossEntropy, displaying the lowest accuracy, indicating they are less suitable for this task. The combination of ConvNeXt-Large with the GAM attention mechanism and FocalTverskyLoss loss function provides the best performance, effectively handling imbalanced data and complex tasks. To more clearly illustrate the performance of different model combinations in terms of average accuracy, the following is a comparison table of each model combination (as shown in Table 3), including the advantages and disadvantages.

**Table 3:** Comparison of performance of various model combinations

Model combination	Average accuracy	Advantages	Shortcoming
ConvNeXt-Large + GAM + FocalTverskyLoss	0.4825	Strong robustness	Lower initial learning speed
ConvNeXt-Large + EMA + FocalTverskyLoss	0.4796	Strong robustness	Lower initial learning speed
ConvNeXt-Large + GAM + DiceLoss	0.4144	Moderate stability	Lower final accuracy
ConvNeXt-Large + GAM + CrossEntropy	0.4114	Moderate stability	Lower final accuracy
ConvNeXt-Large + EMA + DiceLoss	0.3947	Moderate stability	Lower final accuracy
ConvNeXt-Large + MSDA + CrossEntropy	0.3634	Moderate stability	Low accuracy
ResNet50 + ASPP + CrossEntropy	0.1572	Fast initial learning speed	Low accuracy
ResNet101 + ASPP + CrossEntropy	0.1482	Fast initial learning speed	Lowest accuracy

By comparing the performance of different model combinations in terms of average accuracy, it can be seen that ConvGAM model, the one using ConvNeXt-Large as the backbone network and combining with the Global Attention Mechanism (GAM) and the FocalTverskyLoss loss function, is the optimal model. This combination performs well in handling imbalanced data and complex tasks. It has high convergence and stability, making it the best choice for detecting rice blast disease.

### Model performance

**Fig. 8:**
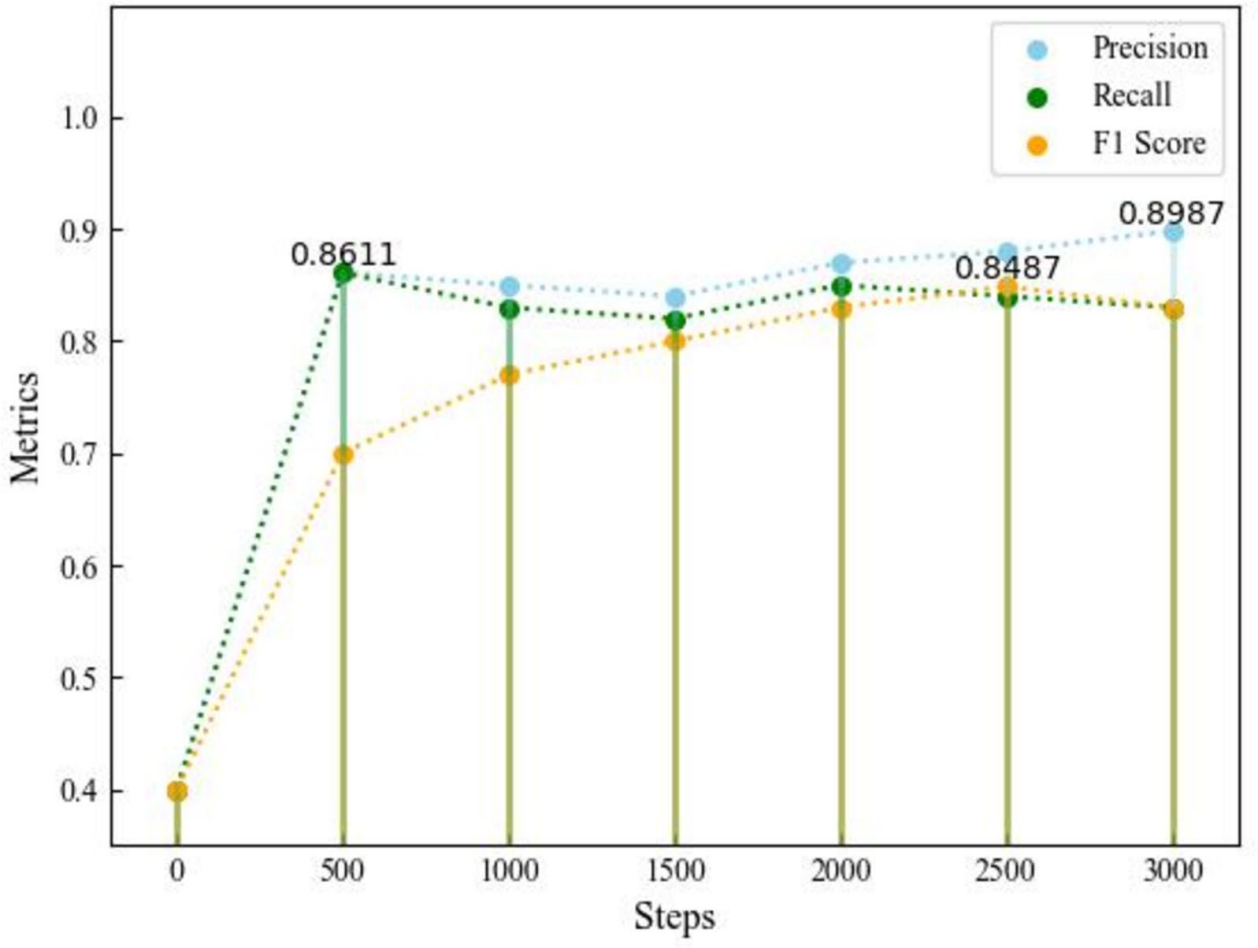
Average Precision, Recall, and F1 Score for the training set. The figure illustrates the trend of precision, recall, and F1 score stabilizing at high levels with increased training steps, based on the training dataset, demonstrating the ConvGAM model’s effectiveness in detecting panicle blast disease, particularly on imbalanced datasets, with further potential for optimization and data augmentation

We conduct a detailed evaluation of the ConvGAM model, which uses ConvNeXt-Large as the backbone network and combines with the Global Attention Mechanism (GAM) and the FocalTverskyLoss loss function. By monitoring the training process of the model, we obtain the average precision, recall, and F1 score at different training steps, as shown in Fig. 8. We calculate the average precision, recall, and F1 score for each disease level category. The changes in these metrics during the training process are as follows:

1. Average Precision: It reaches the highest value of 0.8987 at the 3000th training step.
2. Average Recall: It reaches the highest value of 0.8611 at the 450th training step.
3. Average F1 Score: It reaches the highest value of 0.8487 at the 2400th training step.

The figure shows the trend of each metric with the number of training steps, and also marks the maximum value of each metric. From the figure, it can be seen that as the number of training steps increases, the model's precision, recall, and F1 score gradually stabilize and reach a high level at the end of the training, indicating good performance of the model. Using FocalTverskyLoss as the loss function effectively improves the model's performance on imbalanced datasets, particularly enhancing the accuracy of small sample classes. By introducing the GAM module, the model's ability to focus on global features is enhanced, thus improving feature extraction. From the figure, it is evident that the model's metrics gradually converge during training, indicating a stable training process and good generalization ability. Through the evaluation of performance indicators, the ConvGAM model is shown to perform excellently in detecting panicle blast disease in rice, accurately identifying and classifying different categories of diseased areas. Future work can further optimize the model's parameter settings and explore more data augmentation methods to achieve even better detection results.

### Model test

**Fig. 9:**
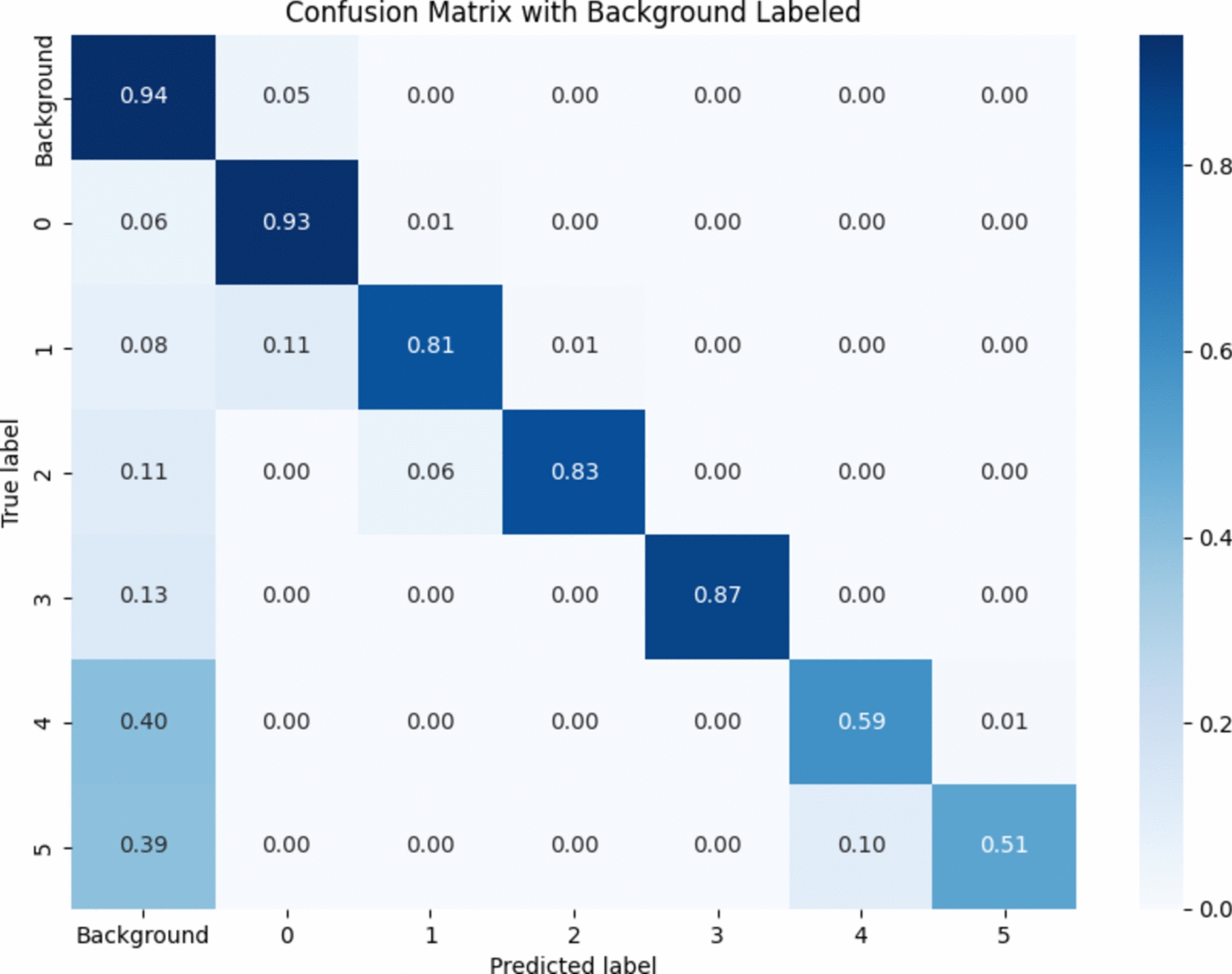
Confusion matrix generated from testing. This confusion matrix shows the performance of the model in classifying different disease levels, where higher diagonal values indicate better classification accuracy, and off-diagonal values represent misclassifications

We used the model trained with the combination of ConvNeXt-Large, GAM, and Focal Tversky Loss, obtained at the 3000th epoch. The test set was used to test the model and generate the confusion matrix. (as shown in Fig. 9). The results show that the model performs well in classifying categories 0, 1, 2, and 3, with accuracies above 0.80 for these categories. However, the performance for categories 4 and 5 is poorer, with accuracies below 0.60, indicating a higher rate of misclassification for these categories. This is because most panicle blast infections are mild or moderate during the initial data collection, with fewer severe infections. Consequently, the data for categories 4 and 5 are limited, leading to data imbalance.

**Fig. 10:**
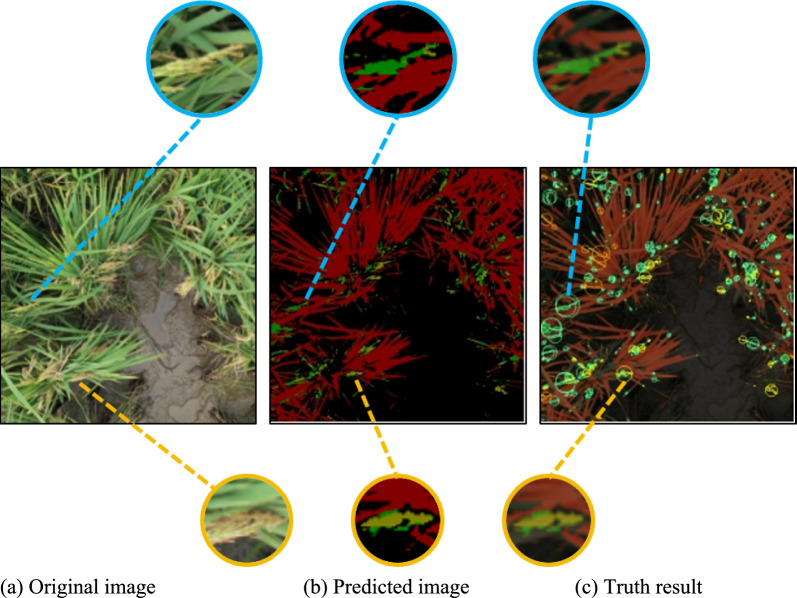
The image of test. The segmentation and detection of diseased areas by color and circles representing different disease levels highlight the strong correlation between the model's predictions and actual results, demonstrating the accuracy and reliability of the ConvGAM model

Based on the model's test accuracy, we conduct validation tests on the image data of panicle blast disease and evaluate the model's performance by calculating the diseased area. We use a set of test images (as shown in Fig. 10), which includes the original image (as show in Fig. 10a), the predicted image, and the diseased area identification image.

**Fig. 11:**
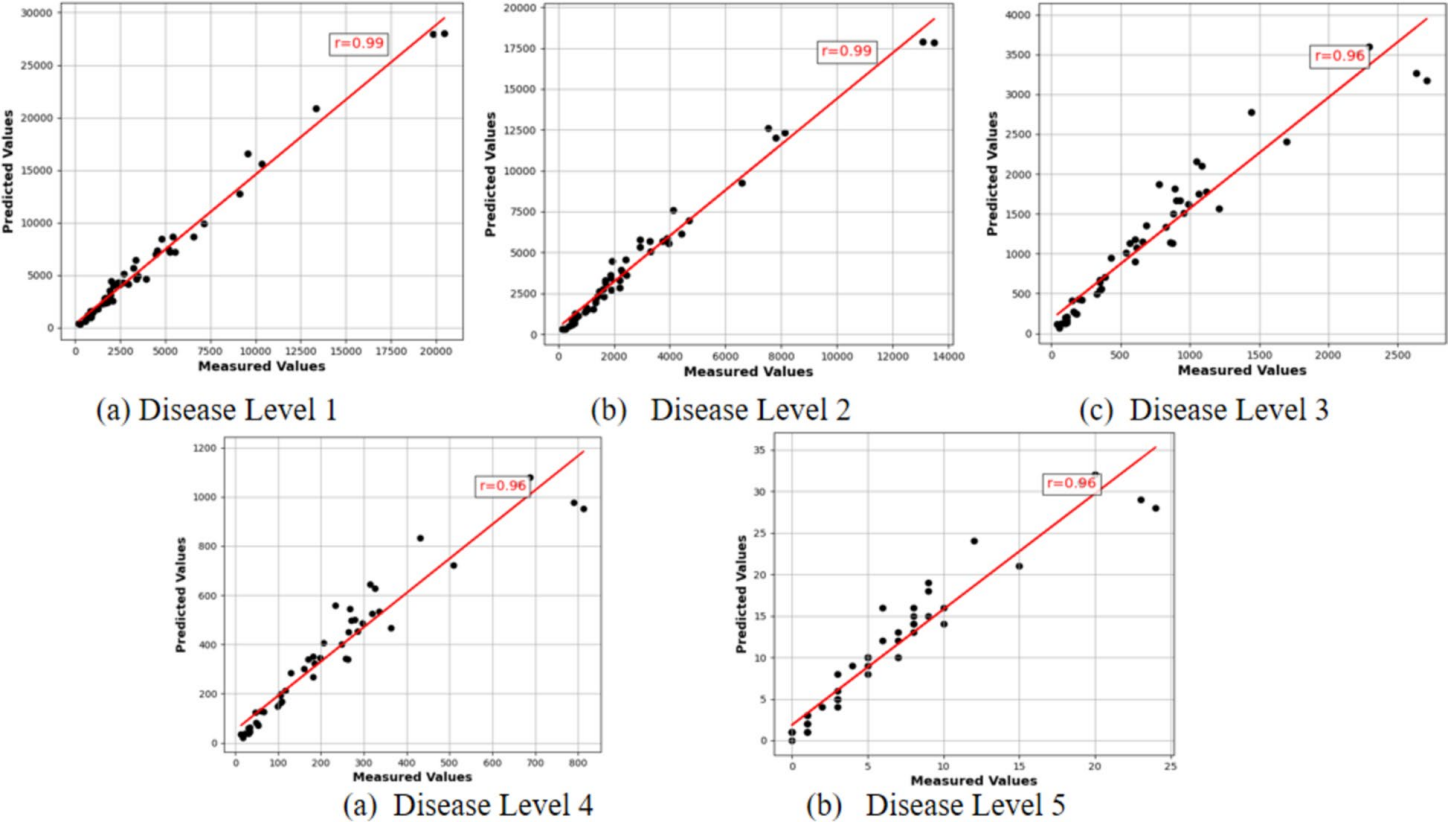
Correlation between predicted and measured values for different disease levels. The scatter plots illustrate a strong correlation between predicted and Measured values across different disease levels, with high correlation coefficients ranging from 0.96 to 0.99, demonstrating the accuracy of the ConvGAM model in predicting diseased areas

In Fig. 10b, different disease level areas are segmented using different colors. Red indicates no disease, mainly the leaf area; green indicates disease level 1; light green indicates disease level 2; yellow-green indicates disease level 3; orange indicates disease level 4; and yellow indicates disease level 5. In Fig. 10c, yellow and green circles are added to mark the detected diseased areas. The color of the circles corresponds to different disease levels, and these circles indicate the areas detected by the model, with the size of the circles representing the extent of the diseased areas. To validate the accuracy and reliability of the model, we also analyze the correlation coefficients to intuitively assess the match between the model's predictions and actual results (as shown in Fig. 11). The correlation coefficient plays a crucial role in evaluating the model's predictive performance. It quantifies the linear relationship between the predicted and actual values, providing a clear metric for the model's accuracy and reliability.

The figure above presents scatter plots, which illustrates the correlation between the predicted counts and the actual counts for different disease levels of rice panicle blast, as well as their respective correlation coefficients. The high correlation coefficients across all levels, ranging from 0.962 to 0.993, demonstrate that the ConvGAM model performs exceptionally well in predicting the counts of diseased areas for various levels of rice panicle blast. The scatter plots show that for Levels 1 and 2, most data points lie close to the ideal correlation line, indicating high accuracy. For Levels 3 to 5, while the data points are more dispersed, the correlation coefficients still indicate strong positive correlations, suggesting the model's predictions are relatively accurate. Overall, the model maintains a high level of accuracy and reliability. Particularly for the lower disease levels, the correlation coefficients for the higher levels are lower, indicating the potential areas for further optimization and enhancement.

### The detection and segmentation performance of the model

**Fig. 12:**
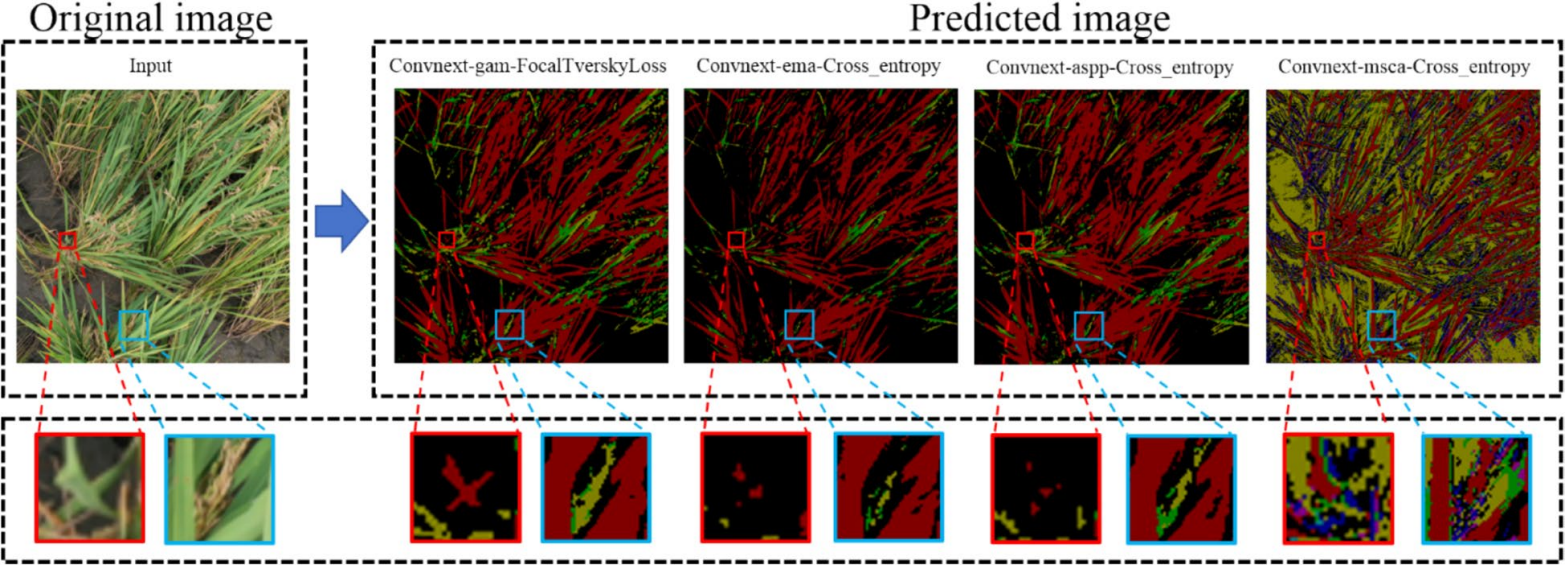
Comparison of model detection effectiveness across different disease levels. The ConvGAM model. Different colors are used to represent various disease levels detected on the rice panicles: red indicates no disease, green represents disease level 1, light green corresponds to disease level 2, yellow-green to disease level 3, orange to disease level 4, and yellow to disease level 5

To further illustrate the detection effectiveness of our model, Fig. 12 shows the process of detecting rice panicle blast disease, including the original image, segmentation results, and highlighted diseased areas.

In Fig. 12, it illustrates the process of detecting rice blast disease on rice panicles using the ConvGAM model. The leftmost panel shows the original RGB image of rice panicles, highlighting regions of interest (ROIs) with red and blue boxes. It indicates the close-up areas for detailed analysis. The subsequent panels display the segmentation results from different models, including the ConvGAM model. Different colors are used to represent various disease levels detected on the rice panicles: red for no disease, green for disease level 1, light green for disease level 2, yellow-green for disease level 3, orange for disease level 4, and yellow for disease level 5. The bottom row contains zoomed-in views of the red and blue boxed areas from the original image, allowing for detailed observation of the segmentation accuracy and the models' ability to identify and classify different levels of infection. This visual representation demonstrates that the effectiveness of the ConvGAM model is superior to others in identifying and classifying rice blast disease in field conditions, which shows its application in agricultural monitoring and disease management.

## Discussion

In this study, we proposed a novel semantic segmentation model, ConvGAM, for detecting rice panicle blast disease. ConvGAM addresses the challenges posed by varying angles, heights, and scales of rice panicles captured in remote sensing images. The model incorporates an enhanced feature extraction mechanism using global attention, allowing for high precision and robustness in feature extraction, which is critical for identifying small and complex disease patterns. The introduction of Focal Tversky Loss further enhances the model's performance on imbalanced datasets, ensuring the accurate detection of less common disease categories.

### Comparison with previous research

In previous research, various methods have been developed to detect rice panicle blast, including multispectral image models, machine learning algorithms (e.g., SVM, Chi-SVM, DBSCAN), and hyperspectral imaging combined with vegetation indices (CVIs). For instance, a multispectral image prediction model based on the linear relationship between grayscale values and disease index achieved rapid, accurate, and non-destructive detection, but its performance on complex and imbalanced datasets was not reported [34]. Similarly, the “spectral bag of words” model using Chi-SVM achieved a classification accuracy of 94.72% for rice blast severity [35], which is slightly higher than ConvGAM's accuracy of 91.4%. However, unlike Chi-SVM, ConvGAM directly addresses the issue of imbalanced datasets and excels in detecting rare or severe disease categories, offering a more balanced performance across all severity levels.

Other studies have utilized deep learning and expert knowledge, such as an improved deep convolutional neural network combined with expert grading, achieving a detection accuracy of 92.0% [36]. While this approach is comparable to ConvGAM in overall accuracy, it heavily relies on manual intervention for expert grading. In contrast, ConvGAM autonomously learns critical features using the Global Attention Mechanism (GAM), enhancing its scalability and applicability to large-scale agricultural datasets.

Additionally, an SVM-based rice panicle blast classification model utilizing small convolutional neural network (CNN) deep features achieved a prediction accuracy of 89.37% [37], which is lower than ConvGAM’s accuracy. This demonstrates ConvGAM's advantage in extracting robust multi-scale features for precise classification. Traditional machine learning models, such as SVM and LDA, also show certain limitations in detecting rice panicle blast, while ConvGAM achieves an overall accuracy of 91.4%, significantly outperforming these methods [38–40]. Although methods combining principal component analysis (PCA), vegetation index (VI), and competitive adaptive reweighted sampling (CARS) with SVM or LDA achieved high classification accuracies ranging from 94 to 97% [41], they still face challenges in handling imbalanced datasets and detecting severe disease categories, which are strengths of ConvGAM. As shown in Table 4, a detailed comparison of ConvGAM with previous methods in detecting rice panicle blast highlights their performance and characteristics.

**Table 4:** Comparison of ConvGAM with previous methods for rice panicle blast detection

Method	Accuracy (%)	Strengths	Weaknesses
Multispectral image model	Not reported	Rapid, accurate, and non-destructive detection	Performance on complex and imbalanced datasets not evaluated
Chi-SVM (“Spectral Bag of Words”)	94.72	High classification accuracy for rice blast severity	Struggles with imbalanced datasets and rare/severe category detection
Improved deep CNN with expert grading	92.0	Comparable accuracy; combines deep learning with expert knowledge	Relies heavily on manual intervention and expert grading
SVM with small CNN features	89.37	Uses CNN deep features to enhance classification	Lower accuracy compared to ConvGAM; limited scalability for large datasets
PCA, VI, CARS + SVM/LDA	94–97	Achieves high accuracy by combining feature extraction techniques	Faces challenges with imbalanced datasets and severe disease category detection
Traditional machine learning (SVM/LDA)	< 91.4	Widely used and interpretable	Limited performance on imbalanced datasets and complex patterns
ConvGAM (ours model)	91.4	Addresses imbalanced datasets, excels at rare/severe disease detection, incorporates GAM and Focal Tversky Loss for robustness, and autonomously learns features	Accuracy slightly lower than Chi-SVM or PCA-based methods in some cases

In summary, ConvGAM not only matches or surpasses the accuracy of many existing methods but also effectively addresses the challenges of imbalanced datasets and rare disease category detection, providing a more comprehensive and robust solution for rice panicle blast detection.

### Practical value

ConvGAM demonstrates strong practical value for agricultural disease detection, especially in large-scale farming environments. Its ability to accurately detect and classify rice panicle blast disease at various severity levels makes it highly suitable for real-time monitoring and intervention in the field. By accurately identifying disease-prone areas, farmers can apply targeted treatments, reducing pesticide use and improving crop yield and quality. Moreover, ConvGAM's robustness in handling imbalanced datasets further enhances its utility, ensuring reliable detection across different disease stages. UAV-captured imagery allows for large-scale monitoring, making the method scalable and efficient for practical agricultural applications.

## Limitations

Despite the promising results, ConvGAM has certain limitations. The model’s reliance on high-resolution UAV imagery makes it sensitive to variations in image quality caused by environmental factors, such as wind and lighting conditions, which could affect the accuracy of disease detection. Additionally, there is still room for improvement in the model’s classification accuracy, particularly in challenging conditions. Future research should focus on developing strategies to mitigate these environmental effects and enhance the model’s robustness and accuracy across diverse conditions.

### Future improvements

Several avenues for future improvement can be pursued to further enhance ConvGAM's performance. First, optimizing the model's hyperparameters such as learning rate and weight initialization could improve its convergence speed and accuracy. Second, more advanced data augmentation techniques, such as generative adversarial networks (GANs) for synthetic data generation, could be explored to increase the diversity and quantity of training samples, particularly for underrepresented disease categories. Finally, integrating additional modalities, such as multispectral or hyperspectral imaging, into the model could improve its ability to capture more complex disease patterns, further enhancing its precision in disease detection.

## Conclusion

The proposed ConvGAM model, by combining the ConvNeXt-Large backbone network with the Global Attention Mechanism (GAM), achieves efficient feature extraction and high-precision detection of rice panicle blast disease. The introduction of Focal Tversky Loss demonstrates excellent performance in handling imbalanced datasets, significantly improving the detection accuracy for less common disease categories. The correlation coefficient analysis for different disease levels shows a high consistency between the model's predictions and actual results, with correlation coefficients of 0.993, 0.989, 0.963, 0.962, and 0.962 for levels 1 through 5, respectively, confirming its reliability. ConvGAM excels in overall accuracy, mean Intersection over Union (IoU), and F1 score, with an overall accuracy of 91.4%, a mean IoU of 0.79, and an F1 score of 0.82. These results indicate that ConvGAM has significant advantages in handling complex and noisy data. Its ability to accurately identify and classify rice panicle blast at various stages of infection makes it a valuable tool for both researchers and farmers. However, further improvements are needed to address the challenges posed by data imbalance and environmental variability, ensuring even more reliable performance in real-world applications.

## Data Availability

No datasets were generated or analysed during the current study.
